# Progressive resistance and flexibility exercises versus usual care advice for improving pain and function after distal radius fracture in adults aged 50 years or over: protocol for the WISE randomized superiority trial

**DOI:** 10.1302/2633-1462.67.BJO-2024-0227

**Published:** 2025-07-08

**Authors:** David J. Keene, Juul Achten, Ioana Marian, Marloes Franssen, Elizabeth Tutton, Warren Sheehan, Colin Forde, Hannah Crook, Jenny Gould, Richard Grant, Duncan Appelbe, Sarah E. Lamb, Matthew L. Costa

**Affiliations:** 1 Exeter Medical School, University of Exeter, Exeter, UK; 2 Nuffield Department of Orthopaedics, Rheumatology and Musculoskeletal Sciences, University of Oxford, Oxford, UK; 3 Oxford Clinical Trials Research Unit, Nuffield Department of Orthopaedics, Rheumatology, and Musculoskeletal Sciences, University of Oxford, Oxford, UK; 4 Patient and Public Involvement Member, Oxford, UK

**Keywords:** Distal radius fracture, Rehabilitation, Clinical trial, Protocol, Exercise, fractures of the distal radius, exercise programme, Upper Extremity, randomized controlled trial, Wrist, resistance exercises, Wrist Injury, grip strength, stiffness, radius fractures

## Abstract

**Aims:**

Distal radius fractures are very common injuries; the majority affect females aged 50 years or over. Most patients experience pain and stiffness in their wrist and upper limb weakness, making activities of daily living difficult. The aim of the WISE (Wrist Injury Strengthening Exercise) trial is to assess the effectiveness of a flexibility and resistance exercise programme for the upper limb compared with usual care advice after distal radius fracture.

**Methods:**

This is a multicentre, parallel-group, superiority, individually randomized controlled trial. We aim to recruit 588 participants aged 50 years and older with a distal radius fracture treated surgically or non-surgically from at least 15 UK NHS hospitals. Participants will be randomized 1:1 using a web-based service to usual care advice plus a therapist-supervised exercise programme (three one-to-one therapy sessions of tailored advice and prescribed home exercise over 12 weeks) or usual care advice only. The primary outcome is participant-reported wrist-related pain and function six months after randomization, measured by the Patient-Rated Wrist Evaluation. Secondary outcomes at three and six months measure health-related quality of life, pain, physical function, self-efficacy, exercise adherence, grip strength, complications, and resource use.

**Conclusion:**

This study will assess whether a therapist-supervised exercise programme is more clinically effective than usual care advice for people aged 50 years and older after distal radius fracture. At the time of submission, the trial is currently completing recruitment; follow-up will be completed in 2025 (ISRCTN registry identifier: ISRCTN78953418).

Cite this article: *Bone Jt Open* 2025;6(7):764–784.

## Introduction

Distal radius fractures are an extremely common injury, representing 18% of all fractures seen in UK hospitals.^1^ Most distal radius fractures occur in females aged 50 years or over after reaching out a hand as a protective response to a fall.^2,3^ Initial fracture management is by non-surgical management using splints or casting or, for more complicated fractures, by surgical fixation followed by splints or casting.

Immediately after initial treatment of the fracture, the vast majority of patients experience pain and stiffness in their wrist and develop muscle weakness of the upper limb, making self-care and activities of daily living difficult.^4^ A prospective cohort of 6,000 older women showed that a distal radius fracture increased the odds of having a clinically important long-term functional decline by 48% (odds ratio (OR) 1.48, 95% CI 1.04 to 2.12).^5^ This functional decline is related to ongoing problems with gripping and lifting tasks (e.g. food preparation, carrying shopping, heavy work tasks), especially if the dominant hand is injured.^5,6^

There are 100,000 distal radius fractures a year in the UK, leading to reduced quality of life for patients and significant costs to health and social care systems.^7^ Improvements in upper limb function would have a particularly important impact on the day-to-day lives of older adults after fracture, and given the large numbers of people affected by distal radius fractures, a wider socioeconomic benefit could result.

### Previous studies

Overall, there is insufficient evidence to assess whether the use of resistance exercises improves recovery after distal radius fracture over usual care. The 2016 Cochrane review on rehabilitation for distal radius fractures included 26 trials with methodological weaknesses and concluded the evidence was “insufficient to establish the relative effectiveness of the various interventions used.”^8^ A more recent systematic review, focusing on prescribed exercise after distal radius fracture, found that across nine trials there was insufficient evidence to support referral to therapy to support patients with exercise progression, or to support the use of specific types of exercise, such as resistance training.^9^ Trials had methodological shortcomings, for example, insufficient sample sizes, suboptimal allocation concealment, no outcome assessor blinding, inadequate reporting of interventions, and substantial loss to follow-up. Previous interventions have also not used contemporary evidence-based guidelines regarding volume and load of exercise to optimize the physiological response to resistance exercise.^10^

Recent trials had conflicting findings; Krischak et al^11^ evaluated a relatively intensive intervention of 12 sessions of physiotherapy over six weeks in 48 adults after surgical fixation of the wrist. They found that patients did worse in terms of upper limb function compared to patients exercising independently at home. Conversely, a recent trial from Chile (n = 74), focusing on adults aged over 60 years undergoing conservative management, reported that additional therapy sessions improved short-term upper limb function outcomes.^12^

We have searched the ISRCTN and clinicaltrials.org trials registries. There are two recruiting trials of motor imagery training (NCT01921062, NCT02957240), and another investigating blood flow-restricted exercise in a military population (NCT03056950). These mechanistic studies will not provide evidence on whether people aged 50 years and over after distal radius fracture achieve better upper limb function when offered resistance exercises.

The 2018 Best Practice for Management of Distal Radial Fractures guidance from the British Orthopaedic Association and British Society for Surgery of the Hand concluded that there was insufficient evidence to inform recommendations on what rehabilitation should be offered after distal radius fracture.^13^ This was consistent with high-quality systematic reviews in this area.^8,9^ The recently completed James Lind Alliance Priority Setting Partnership (JLA PSP) for ‘Broken Bones of the Upper Limb in People over 50’ identified rehabilitation as the third highest ranked research priority.^14^

Current guidance for rehabilitation, in the absence of evidence, reflects common practice in the UK, which is advice from a surgeon or a physiotherapist on self-management.^13^ The advice usually includes basic upper limb mobilizing exercises to help recover joint range of motion, and provision of a patient information leaflet. Referral to physiotherapy or specialist hand therapy for supervised rehabilitation and specialist splinting is variable, and often reserved for the minority of patients who experience serious complications.^13^

### Resistance exercise after distal radius fracture

We hypothesize that introducing structured flexibility and resistance exercise training has the potential to improve functional recovery by optimizing recovery of muscle strength of the hand and upper limb.

Current exercise advice focuses on joint flexibility and on graded increase in use of the hand in day-to-day activities. However, reduced muscle strength is also a potentially important issue to target with exercise. Muscle strength has been shown to be more closely related to lower levels of patient-reported upper limb function than range of joint motion after distal radius fracture.^15,16^

Functional deficits after distal radius fracture are related to the capacity to perform heavy gripping, pushing, and lifting tasks.^5,6^ It can take over two years for grip strength to return to levels close to the uninjured limb, and limitations in dexterity (manipulating the wide range of objects people need to interact with in day-to-day life) have been shown to be persistent.^17^ For older adults, these difficulties with daily functional tasks are a particular problem.^18^ For example, patients have long-term difficulties with keys, door handles, carrying, pushing to open doors or to get out of the bath; physically demanding work tasks and gardening are also commonly problematic in the long term. These experiences were echoed by the Patient and Public Involvement (PPI) group, who co-developed this study. This indicates that a resistance exercise programme could address the muscle strength required to perform a wide range of functional movements of the hand and upper limb.

### Supporting recovery from distal radius fracture

Two PPI representatives on the JLA PSP for ‘Broken Bones of the Upper Limb in People over 50’ steering committee were keen to support a new study on exercise after distal radius fracture and have joined the research team as members of the trial management group and as co-applicants, with peer support from the UK Musculoskeletal PPI Group. Our PPI team members and attendees of our PPI event, held in the early stages of developing this protocol, identified that supporting people to exercise after fracture is a very important issue to address. They highlighted experiences of ‘lacking confidence’ after injury, and ‘fearing that exercises could be damaging’, particularly as they are ‘often sore’. Older age and greater pain after injury have recently been identified as prognostic factors that reduce the chances of regaining upper limb function after distal radius fracture.^19^ However, psychological factors such as anxiety and catastrophic thinking (a focus on worst-case scenarios) are also consistently found to be important predictors of upper limb function recovery.^20^ In line with this, non-adherence to outpatient prescribed exercise for musculoskeletal problems is estimated to be as high as 70%.^21^ Risk factors for low adherence include low self-efficacy (confidence in own ability to achieve an outcome), anxiety, and greater perceived barriers to exercise.^22^

Some of these risk factors for poor recovery and exercise adherence are modifiable in the context of a therapy intervention. However, existing clinical trials of distal radius fracture rehabilitation have not targeted pain beliefs and psychological barriers to resistance exercise.^9^ This study will draw on evidence from the Strengthening And stretching For Rheumatoid Arthritis of the Hand (SARAH) trial, which evaluated a structured flexibility and resistance exercise programme for people with rheumatoid arthritis of the hand.^23^ Hand and upper limb exercises in addition to usual care were found to be a clinically and cost-effective way of improving function. In the SARAH trial, improvements in self-efficacy to exercise and exercise adherence were achieved by using simple behaviour change strategies. We propose using these strategies, adjusted for patients after distal radius fracture.

In this study, we propose to deliver exercise advice to patients that aims to support engagement in a home exercise programme. In accordance with the Medical Research Council guidance on the development and evaluation of complex interventions,^24^ we assessed the overall adherence, feasibility, and experiences of different levels of face-to-face contact from a physiotherapist or occupational therapist prior to this definitive study. The feasibility phase (NIHR200458) informed the decision on the best mode of delivery to be taken forward to this definite full randomized controlled trial (RCT).

## Methods

### Aims

The aim of this multicentre, parallel-group, individually randomized superiority trial (nonCTIMP) is to compare the clinical effectiveness of a therapist-supervised exercise programme, compared to usual care advice, in improving pain and function after distal radius fractures in adults aged 50 years and over. The primary objectives and outcomes measures are shown in Table I, while the secondary objectives and outcome measures are outlined in Table II.

**Table I.** Primary objectives and outcomes measures.

Objective	Outcome measure	Timepoint(s) of evaluation of this outcome measure	Data required	Source data (including location)
To quantify and draw inferences on differences in wrist pain and function between intervention groups	PRWE	Six months post-randomization	Completed PRWE questionnaire	Participant-reported outcome questionnaire

PRWE, Patient-Rated Wrist Evaluation.

**Table II.** Secondary objectives and outcome measures.

Objective	Outcome measure	Timepoint(s) of evaluation of this outcome measure	Data required	Source data (including location)
To quantify and draw inferences on differences between the intervention groups in:	PRWE	Three months post-randomization	Completed PRWE questionnaire	Participant-reported outcome questionnaire
	PRWE pain subscale	Three and six months post-randomization	Completed PWRE pain subscale in questionnaire	
	PRWE function subscale	Three and six months post-randomization	Completed PWRE function subscale in questionnaire	
	Upper limb function as measured by the PROMIS Physical Function (Upper Limb) score	Three and six months post-randomization	Completed PROMIS questionnaire	
	Health-related quality of life as measured by EQ-5D-5L	Three- and six-months post-randomization	Completed EQ-5D-5L questionnaire	
	Confidence in ability to exercise as measured by the SEE scale	Three and six months post-randomization	Completed SEE questionnaire	
	Exercise adherence measured by self-reported exercise frequency	Three and six months post-randomization	Completed adherence questionnaire	
	Upper limb muscle strength as measured by grip strength (cylindrical grip)	Six months post-randomization	Dynamometer readings	Readings taken and recorded by participant
	Adverse events-related complications, as measured by patient questionnaires and site complications	Three and six months post-randomization	Completion of participant questionnaire; completion of complications report by site	Participant-reported outcome questionnaire; site complications report
	Health resource use as measured by self-reported bespoke questionnaire (including primary and secondary care consultation, additional therapy, investigations, surgery, prescribed and over-the-counter pain medication), sick leave (days), out-of-pocket expenses	Three and six months post-randomization	Completion of participant questionnaire	Participant-reported outcome questionnaire

EQ-5D-5L, EuroQol five-dimension five-level questionnaire; PROMIS, Patient-Reported Outcome Measurement Information System; PRWE, Patient-Rated Wrist Evaluation; SEE, Self-Efficacy for Exercise scale.

### Exploratory objectives/additional mechanistic objectives and outcomes

There are no additional exploratory/mechanistic objectives and outcomes in this study.

### Primary outcome/justification for the follow-up period

The primary outcome is the Patient-Rated Wrist Evaluation (PRWE)^25^ at six months post-randomization, a 15-item patient-reported questionnaire that assesses pain and functional difficulties in activities of daily living resulting from injuries affecting wrist joint area (total score ranges from 0 to 100, higher scores indicate worse wrist pain and function). The pain subscale has five items (0 = no pain, 10 = worst pain) and the function subscale has ten items (0 = no difficulty, 10 = unable to do). A total score on a scale of 100 is calculated from the two subscales equally weighted (0 = no disability). The total score is the trial primary outcome, with the two subscales also reported as secondary outcomes.

It is anticipated that if the intervention is effective compared to usual care advice, then improvements in pain and function will be observed within the first six months, hence the trial follow-up period.

The Core Outcome Measures in Effectiveness Trials (COMET) guidance for distal radius fractures identified pain and function as the primary domains for outcome measurement, and recommended the PRWE.^26^

### Secondary outcomes

- Wrist pain and function measured using the PRWE at three months post-randomization, as described for the primary outcome above.
- Wrist pain subscale measured using the PRWE at three and six months post-randomization.
- Wrist function subscale measured using the PRWE at three and six months post-randomization.
- Upper limb function assessed using the Patient-Reported Outcome Measurement Information System (PROMIS) Physical Function (Upper Limb):^27^ PROMIS questionnaires are administered electronically. They are a computer-adaptive test, which are dynamic tests based on item response theory. A mathematical model adapts the sequential questions asked based on a participant’s previous response. A tailored set of questions is therefore asked from a large item pool. PROMIS instruments are scored from 0 to 100, with 50 points representing the mean score for the USA general population; higher scores indicate better function. This questionnaire has been found to be valid in the context of upper limb fractures in the UK.^28,29^ Participants with no internet access will be able to complete a paper-based version of the PROMIS questionnaire (PROMIS Physical Function Upper Extremity Short Form, 7 a).
- Self-efficacy to exercise measured using the Self-Efficacy for Exercise scale (SEE):^30^ a nine-item participant-reported questionnaire (total scores range from 0 to 90, higher scores indicate higher self-efficacy for exercise) will be used to assess the participants’ confidence in their ability to exercise.
- Exercise adherence: Participants will be asked to indicate how many times in the preceding week they have done specific exercises for their injured hand and upper limb, to assess engagement with the advised exercises after wrist fracture.
- Quality of life measured using the EuroQol five-dimension five-level questionnaire (EQ-5D-5L), a validated, generalized and standardized instrument comprising a visual analogue scale (VAS) measuring self-rated health and a health status instrument, consisting of a five-level response (no problems, some problems, moderate problems, severe problems, and unable) for five domains related to daily activities: 1) mobility; 2) self-care; 3) usual activities; 4) pain and discomfort; and 5) anxiety and depression.^31^ Responses to the health status classification system are converted into an overall score using a published utility algorithm for the UK population. The EQ-5D health status scale ranges from negative scores −0.594 (reflective of a patient’s quality of life being worse than death), 0 (death), to 1 (perfect health). A respondent’s EQ-VAS gives self-rated health on a scale where the endpoints are labelled ‘best imaginable health state’ (100) and ‘worst imaginable health state’ (0).
- Health resource use: A bespoke health resource use questionnaire will be developed assessing number of primary and secondary care consultations, additional therapy, further wrist adiographs and scans, surgery, prescribed and over-the-counter pain medication, out-of-pocket expenses, and work absence.
- Adverse events (AEs): All unexpected serious adverse events (SAEs) related to the randomized interventions will be recorded. These events will be reported by recruitment centres as they become aware of events. Foreseeable adverse events will be recorded as complications both by recruitment centres and participants.
- Muscle strength of the upper limb: Force produced during cylindrical grip (for powerful grasping tasks and holding handles) will be measured using a commercially available hand-held dynamometer.^32,33^ The participant is asked to produce the maximum force that they can manage in a set starting position in sitting with the arm by the side and elbow bent to 90° in mid-prone. Participants start with one attempt on the uninjured side then one attempt on the injured side. This sequence is repeated three times so that three attempts are recorded for each hand. The best score for the injured side is compared to the best score for the uninjured side. Participants are asked to wait a minimum of 30 seconds between recording measurements. Participants will also be asked to repeat the strength assessments on the next day; if they are willing to do so, this second attempt will be optional.

## Study design and setting

WISE (Wrist Injury Strengthening Exercise) is a multicentre, parallel-group, superiority RCT assessing therapist-supervised exercise versus usual care for adults aged 50 years or over after distal radius fracture with direct patient follow-up, up to six months.

**Fig. 1:**
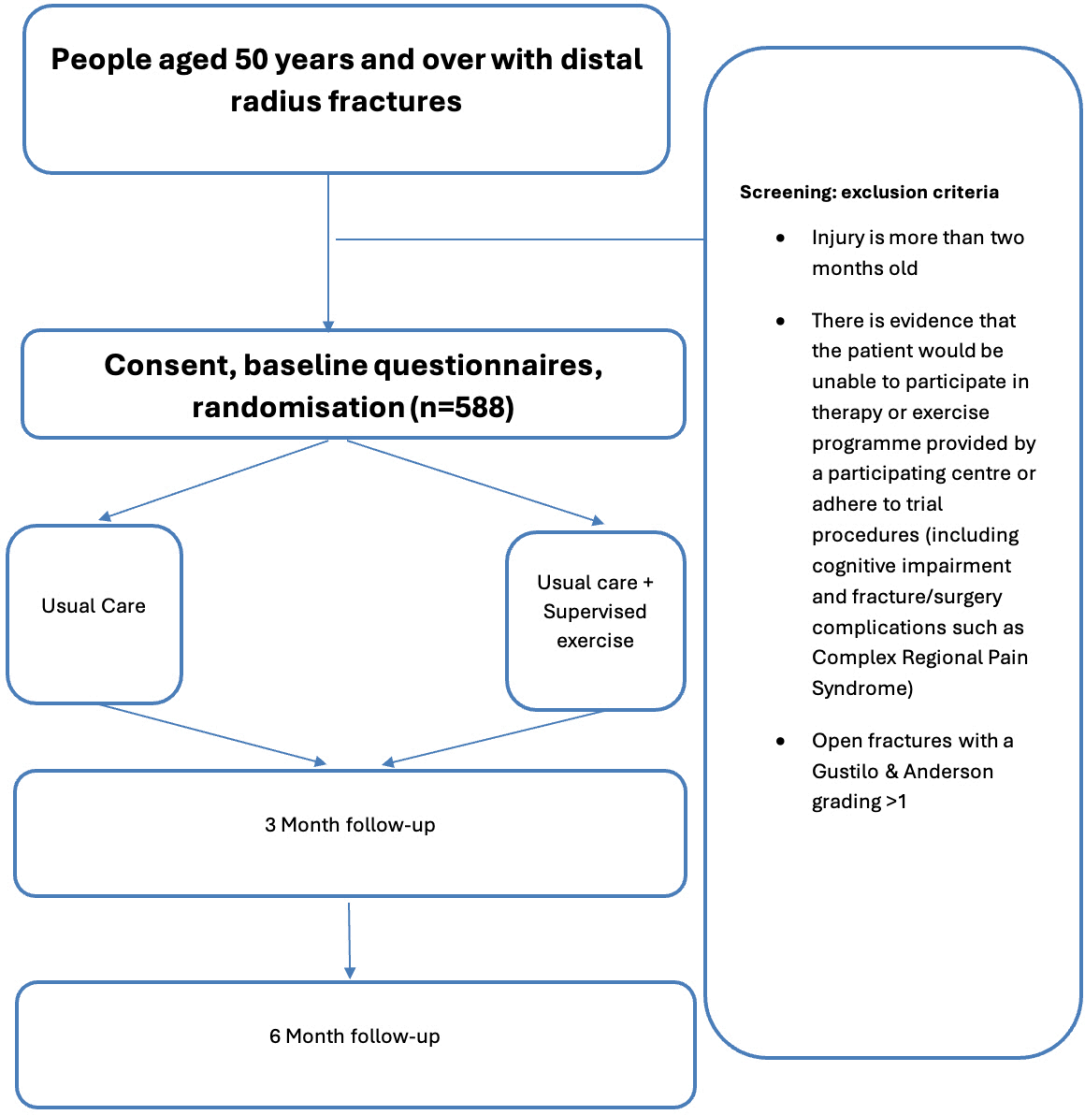
Study flowchart.

The trial will recruit 588 patients (294 in each of the two arms) with a distal radius fracture from approximately 15 NHS sites in the UK. Participants will be individually randomized (1:1) to receive usual care advice or therapist-supervised exercise in addition to usual care advice. Exercise and advice are standard NHS treatments and will be conducted by the recruiting centres. Participants will be followed up clinically as per standard hospital policy. They will be followed up via electronic questionnaires by the central study team at three and six months post-randomization. If the participant does not have internet access, they will be sent a postal questionnaire by the central study team. After completion of the six-month questionnaire participants will also be sent a handheld dynamometer in the post to complete grip strength measurements. The results of the measurements posted back by the participants will be recorded electronically on the study database. A study flowchart is provided in Figure 1.

### Recruiting sites/site types

Participants will be recruited from the emergency department (ED)/minor injuries units or via virtual or outpatient trauma and orthopaedic services (fracture clinic) from approximately 15 NHS hospital trauma departments and related therapy teams who see patients with distal radius fractures.

### Collection of outcome data and follow-up assessments

Participants will receive communications from the central study team to collect outcome data. For those who consent to take part in the WISE study, the following information will be recorded on a secure web-based form in the WISE study database system REDCap (Research Electronic Data Capture)^34,35^ by the attending member of the research team.

For those who consent to take part in the WISE study, the following information will be recorded on a secure web-based form in the WISE study database system REDCap by the attending member of the research team. To enable follow-up, participant details will be collected (name, address, NHS/CHI number, date of birth, telephone number, mobile number, email address, general practitioner (GP) name, and GP address).

The GP details are required to allow the central study team to send a letter to the participant’s GP to inform them of their WISE study participation. The email address will enable a copy of the completed consent form to be sent to the participant or, at their request, a different individual for safekeeping. Depending upon participant preference, the email/postal address and/or telephone number may be used for follow-up questionnaires and reminders/text messages.

### Participant involvement

All recruiting sites are in the UK. Participants will be in the study for approximately six months from randomization to last protocol contact. Following a participant’s final protocol contact, they will receive standard care.

### Expected recruitment rate

The anticipated monthly recruitment rate is four participants per month per recruiting site. It has been estimated that 50% of eligible patients will consent to take part in the study. It is also anticipated that all sites will be open within seven months of starting recruitment.

### Equality, diversity, and inclusion for study participants

The INCLUDE framework and the key questions worksheet developed by Trial Forge guide researchers to carefully consider key under-served populations in their specific field.^36^ In the case of trauma research, we consulted with PPI partners and clinical stakeholders at centres with diverse catchment populations. It was evident from this work that people from more deprived areas with lower educational levels are the key group that can be less engaged with health services and research participation after trauma. We have included a range of procedures to ensure inclusion from this key underserved community, which are highlighted here:

- Use of heat maps of regional sociodemographics and historical research activity to aid site recruitment planning.
- An explainer video will augment written patient information sheets.
- A dedicated member of the research team will be available for the patients and their families to answer questions and reassure them about taking part in the study.
- Information presented to potential participants and their families has been developed by the clinical members of the trial team in close collaboration with our PPI co-applicants, who have relevant lived experience.
- All web resources will undergo significant user testing and have adjustable font sizes, and all videos will have voiceovers and subtitles.
- We will ensure there is a postal option for follow-up to enable those with fewer IT skills/less access to participate.
- Screening data will be reviewed each month by the trial management team to assess whether representative samples of patients are being approached, and to ensure no selection bias occurs in any of the centres with regard to approach and inclusion/exclusion of specific groups of patients. Screening logs recording the age, sex, initial fracture management (surgical or non-surgical), deprivation index, ethnicity, and, if provided, the reasons for declining participation will be kept at each site to determine the number of patients assessed for eligibility and reasons for exclusion.
- Training of site staff on accurate and inclusive screening and recruitment will be through newsletters, regular Q&As/top tips, and refresher sessions.

### End of study

The end of study is the point at which all case report form (CRF) data relating to the study primary and secondary outcomes has been entered/received and all queries resolved. The study will stop randomizing participants when the stated number of patients to be recruited is reached.

The sponsor and the chief investigator reserve the right to terminate the study earlier at any time. In terminating the study, they must ensure that adequate consideration is given to the protection of the participants’ best interests.

## Participant eligibility criteria

Eligibility will be assessed upon initial entry into the study and confirmed at the point of randomization.

### Overall description of study participants

Written informed consent must be obtained before any study-specific procedures are performed. Participant eligibility will be confirmed by a suitably qualified and experienced individual who has been delegated to do so by the principal investigator (PI) based on the below criteria.

### Inclusion criteria

A patient will be eligible for inclusion in this study if all of the following criteria apply:

- Aged 50 years or over with a distal radius fracture treated surgically or non-surgically
- Willing and able to give informed consent

### Exclusion criteria

A patient will not be eligible for the study if any of the following apply:

- Injury is more than two months old
- There is evidence that the patient would be unable to participate in therapy or a self-guided exercise programme provided by a participating centre or adhere to study procedures (including cognitive impairment and fracture/surgery complications, such as complex regional pain syndrome)
- Open fractures with a Gustilo & Anderson grading > 1

### Rationale for inclusion and exclusion criteria

After the age of 50 years, bone mineral density decreases steadily in males, while in females there is an initial decline between aged 50 and 65 years, with a further decline in the age groups thereafter.^37^ In the UK incidence of distal radius fractures climbs rapidly from aged 50 years, more so for females.^2^ Previous studies provide strong evidence that people aged 50 years and over become increasingly vulnerable to fragility fractures of the distal radius.

Patients who have contraindications to the study intervention or are not suitable for usual care advice (for example, due to fracture-related complications) will not be eligible. Patients with more severe open fractures are also not eligible, as these rarer injuries usually have different rehabilitation pathways.

### Protocol waivers to entry criteria

Protocol adherence is a fundamental part of the conduct of a randomized clinical trial. There will be no waivers regarding eligibility (i.e. each participant must satisfy all the eligibility criteria). Changes to the approved inclusion and exclusion may only be made by a substantial amendment to the protocol.

Before entering a patient onto the study, the PI or designee will confirm eligibility. If unsure whether the potential participant satisfies all the entry criteria, and to clarify matters of clinical discretion, investigators should contact the central study office, who will contact the CI or designated clinicians as necessary. If in any doubt, the CI must be consulted before recruiting the patient. Details of the query and outcome of the decision must be documented in the investigator site file (ISF)/trial master file (TMF).

## Screening and recruitment

### Participant identification

Participants will be recruited from the ED/minor injuries unit or virtual or outpatient trauma and orthopaedic services (fracture clinic) in NHS hospitals in the UK. The following methods will be used to identify potentially eligible participants:

- Searching of clinic records/hospital databases by the usual care team to identify individuals who may be eligible to enter the study
- Identification during routine clinic visits

Potentially eligible patients will be identified by searching of clinic records/hospital databases at participating research sites by those in the clinical care team only (who may also be a member of the site research team). Any patients who are thought to fulfil the inclusion/exclusion criteria will be offered a patient information sheet (PIS). As per the feasibility phase of the study, the PIS and the explainer animation video will outline the treatments being compared (usual care advice compared with supervised exercise (three therapy sessions)), but will not detail the specific types of exercise being evaluated (flexibility and resistance exercises). The aim of this is to reduce contamination between the treatment groups.

Potentially eligible patients identified during routine clinic visits will be provided with a PIS by a member of their usual care team (who may also be a member of the site research team) and asked to consider the study. Where their usual care clinician is not a member of the site research team, potential participants will be asked if it would be acceptable for their name and contact details to be passed to the site research team who will make contact at a later timepoint (this may be in person in a clinic or via telephone or video call in accordance with local site practice) or during a further routine clinic visit, or potential participants may be given the PIS and asked to call the number on it if they wish to find out more about the study. When a potential participant is approached for permission, for their details to be passed onto the site research team; if this permission is given, this should be recorded in their clinical notes.

### Use of screening logs

A screening log (within the REDCap study database) will be used to record information about the number of patients considered and/or approached for the study and if provided, the reasons for declining participation. The following personal data are collected on the screening log: age, sex at birth, deprivation index, and ethnic group. A screening number will be assigned to each patient screened. Screening data will be reviewed by the trial management team to assess sample representativeness and to ensure no selection bias occurs with regard to approach and inclusion/exclusion of specific groups of patients.

## Study intervention and comparator

### Supervised exercise in addition to usual care (intervention)

Participants will receive usual care, and then a single physiotherapy or occupational therapy session of up to 60 minutes on the same day as their clinic visit, or the next available appointment. The session has to take place as soon as possible and no later than three weeks after the cast/splint removal. The purpose of this session will be to assess the participant and introduce the self-managed flexibility and resistance home exercise programme. Participants will then have two individual follow-up therapy sessions of up to 30 minutes over 12 weeks after the first session. If the hospital has pre-established practices for remote video or telephone consultations, then the participant will be able to access this mode of delivery.

Over the sessions there will be opportunities for feedback to participants on the progress of their rehabilitation: this will include reinforcement during the sessions, each having a strong evidence base to support their use in older adults.^38^ Therapists will focus on helping participants identify barriers to exercise and facilitate problem-solving over the sessions.

The exercise programme will be based on the SARAH trial intervention,^23^ which is a highly structured system of hand and upper limb exercises. The programme will enable participants to progress their exercises after the initial set-up session with the therapist. The programme will utilize a range of resistance exercises based upon functional movements designed to promote recovery of the strength required for activities of daily living (e.g. chopping, lifting, pushing, jar-opening).

It is widely accepted in the field of exercise physiology that an increase in strength requires a sufficient training stimulus in terms of volume and intensity: this is called the overload principle.^10^ The previous successful resistance training interventions developed and evaluated by our team have highlighted that the duration of a programme, the specificity of exercises, and individualization (i.e. adjusting the programme to suit each participant) are important considerations for success.^39^ Progression is also required to maintain improvement and prevent potential reversal of training effects.

Participants will be provided with the required equipment for the exercises, such as resistance bands and exercise putty, as part of the study. The exercises will be rehearsed with the therapist and then practised at home over at least 12 weeks. This time period allows sufficient time for neuromuscular adaptation to take place.^40^

Our PPI group highlighted the challenges of motivation when exercising independently, and that the quality of information would be crucial to success. Self-management support materials are in the form of a workbook containing exercise advice. A high-quality workbook is important, as there is evidence that patients do not retain much of the information that they are provided with face-to-face.^41^ An exercise diary contained within the workbook will support exercise programme adherence. The intervention will require therapists to offer education about the potential benefits of resistance exercise for the participants in achieving their personal goals, managing pain, and to reassure them about their capacity to exercise.^42^

The combination of exercise adherence techniques from the SARAH trial will be used.^23^ The intervention draws on the evidence-based NHS Health Trainers Handbook.^43^ These practical and easy-to-use techniques have been used in several other physiotherapy studies conducted within our group.^23,44,45^ The techniques utilize a two-stage mechanism: 1) increasing intention to adhere to the exercise regimen; and 2) enabling translation of this behavioural intention into actual behaviour.

The strategies will involve the participants completing a written plan. The plan includes identifying personal goals, making action plans as to where and when the home exercises will be performed, and a contingency plan for how to manage difficulties with the exercises. The participant will sign this document (either on paper or electronically) to confirm for themselves their intention to do the exercises, as there is evidence that this can facilitate engagement in an exercise programme.^46^

Participants will be shown the website and provided with a unique log-in. The website content will mirror the workbook but will contain advice videos, exercise demonstration videos, written information, motivating notifications, and an ability to track progress by using an online version of an exercise diary. We will carefully monitor uptake and use of the online materials. Feedback from our PPI group suggested that the website would be a valued option, as after being shown exercises in a clinic it can be difficult to recall the technique, which could be a potential barrier to doing the home exercise programme.

In line with usual practice, participants who have difficulties with the exercise programme will be supported over the phone/videoconference as per local practice. We will carefully monitor the use of any additional contact required as part of this study.

### Usual care (comparator)

On the day of cast or splint removal (usually four to eight weeks after fracture), advice on self-management will be delivered by a surgeon, physiotherapist, or occupational therapist in a fracture clinic, as per routine clinical practice. Advice will include hand and wrist mobilization exercises to restore flexibility and guidance on building up activity gradually. Participants may also receive a general advice leaflet, as per routine practice at that hospital. Participants in this group will not be provided with access to the intervention materials (the supervised exercise workbook and website).

### Concurrent healthcare

Other aspects of the participant’s healthcare will be as per usual NHS local practice. Participants would be able to access therapy services for post-fracture complications such as development of complex regional pain syndrome according to usual local routes of referral. Use of additional therapy will be carefully recorded during the study. We will collect information on post-randomization additional surgery, wrist radiographs and scans, and use of pain medication to gain an understanding of the costs of the interventions.

## Informed consent

### Consent procedure

Informed consent will be sought, and if a person approached is willing to give consent, it will be obtained by a member of the site research team listed on the delegation log before the potential participant undergoes any study-related procedures or interventions. A member of the site research team will explain the details of the study in addition to the already presented PIS, ensuring that the potential participant has sufficient time to consider whether or not to participate. A member of the site research team (authorized to do so on the delegation log) will answer any questions that the potential participant has concerning study participation.

### Time allowed to decide to take part

Patients will be given as much time as possible to consider the information and discuss it with relatives/carers. Patients will be randomized at the time they have their cast/splint removed (or within three days of this time, if getting in touch via a telephone or video call after the clinic visit), so they will have up until this point to consider the study and decide whether or not to consent. It will be clearly stated that the participant is free to withdraw from the study at any time for any reason without prejudice to future care, without affecting their legal rights, and with no obligation to give the reason for withdrawal. If the patient cannot be contacted within 14 days of the local research team providing the full PIS, their contact information will be removed from REDCap if it has been saved there.

### Completion of the informed consent form

The patient and the investigator (or authorized designee) must personally sign and date the current approved version of the informed consent form.

The ICF will usually be offered to participants in clinic as an electronic form on a tablet device, laptop, or desktop computer (with the consent form being filled in directly on the study database, REDCap). Where it is not possible for a consent form to be completed in clinic (for example, if a participant has only had telephone appointments), remote electronic consent may also be used. Remote eConsent (using REDCap) will be obtained in accordance with the Oxford Clinical Trials Research Unit (OCTRU) standard operating procedure for obtaining consent.

Where consent forms are completed electronically, signatures will be either achieved by a finger tracing across a tablet device, using an electronic stylus on a tablet device, or by using a mouse dragging the cursor across the screen. All methods are to be used as if signing with a traditional pen.

Where electronic consent is used and the participant has an email address that they are willing to provide, an electronic version of the signed ICF will be automatically emailed to them. If the participant does not have or does not provide an email address, the site research team will be able to print a copy of the signed ICF and provide this to the participant. A copy of the electronic consent form downloaded from the study database should be placed in the ISF and a copy placed in the participant’s medical record.

If the participant does not have internet access and is not in a physical clinic, the consent will be recorded by a member of the local team on a verbal ICF during the informed consent discussion via a telephone call. Once completed, this form will be signed by a member of the site research team authorized to do so and then sent via email as a PDF document or by post to the participant, as per their preference.

### Individuals lacking capacity to consent

Individuals lacking capacity to consent to study participation will not be eligible to enter the study. As this is an intervention requiring active self-management, following advice and instructions, and use of written materials, all participants will be required to have the capacity to consent to participation and sufficient cognitive function to manage a home programme.

### GP notification

Permission from the participant will also be obtained to inform their GP of their inclusion in the study. An approved GP letter will be sent by the central study team or site research team together with study information to the participant’s GP, informing them of their participation in the study.

### Re-consenting

Should there be any subsequent amendment to the final protocol which might affect a participant’s participation in the study, continuing consent will be obtained using an amended consent form which will be signed by the participant.

### Participants who lose capacity during the study

In the rare event that a participant who has previously given consent loses capacity, the participant would be withdrawn from the study. Identifiable data already collected with consent would be retained and used in the study. No further data would be collected, nor any other research procedures carried out on or in relation to the participant.

## Randomization

### Randomization procedure

Randomization will take place once eligibility is confirmed, informed consent has been given, and baseline questionnaires have been completed. Participants will be randomized to one of the treatment arms outlined in Table II by the site research team via a centralized validated computer randomization program through a secure (encrypted) web-based service, provided by the OCTRU, accessed via the WISE REDCap study database.

**Table III.** Treatment arms.

Arm	Treatment
Supervised exercise in addition to usual care advice (intervention)	Face-to-face/virtual therapy session of up to 60 mins Two additional therapy sessions up to 30 mins Access to the study-specific workbook and website
Usual care advice (comparator)	Advice on self-management as per local practice

### Randomization methodology

Upon randomization of a participant, the central study office and a member of the site research team will be notified by an automated email. Full details of the randomization procedure will be stored in the randomization and blinding plan in the confidential statistical section of the eTMF.

Consented participants will be allocated randomly (1:1) to either supervised exercise in addition to usual care advice or usual care advice. Randomization will be performed using a minimization algorithm (or randomization schedules) to ensure balance between the two treatment groups for the stratification factors: recruiting centre; and initial fracture management (surgical compared with non-surgical).

In the feasibility phase, the first few participants were randomized using simple randomization, to seed the minimization algorithm, and a non-deterministic probabilistic element was included to prevent predictability of treatment allocation. For the full study, the minimization will be seeded using the participant records randomized during the feasibility stage in the two groups (supervised exercise in addition to usual care advice and usual care advice), and a non-deterministic probabilistic element will be included. The randomization schedule will be designed by the OCTRU study statistician, and full details will be detailed in the randomization and blinding plan.

Justification for stratification factors: Stratification will be used to ensure equal allocation of participant subgroups to the intervention and control groups across important baseline prognostic factors. Stratification factors include recruiting centre to account for potential variability in outcome due to site, and initial fracture management, as surgical compared with non-surgical treatment can be an important prognostic factor.

Table IV shows the scheduled assessments for the study.

**Table IV.** Schedule of assessments.

Assessments	Screening	Baseline	Supervised exercise sessions 1 to 3	3 mths	6 mths
Site completed					
Assessment of eligibility criteria (screening log)	X				
Informed consent	X				
Baseline sociodemographic, handedness, and injury data		X			
Usual care treatment log		X			
Supervised exercise treatment log			X*		
Participant completed					
Informed consent	X				
Baseline sociodemographics		X			
PRWE		X		X	X
PROMIS Physical Function (Upper Extremity)		X		X	X
EQ-5D-5L		X		X	X
SEE		X		X	X
Grip strength measurements					X
Self-reported exercise frequency				X	X
Complications				X	X
Resource use				X	X

* Supervised rehabilitation group only.

EQ-5D-5L, EuroQol five-dimension five-level questionnaire; PROMIS, Patient Reported Outcome Measurement Information System; PRWE, Patient-Rated Wrist Evaluation; SEE, Self-Efficacy for Exercise scale.

### Study questionnaires

Where possible, questionnaires will be completed electronically by the participant. Participants will receive a link via email or text message to complete their study questionnaires (participants will be asked at their baseline visit whether they wish to complete follow-up questionnaires electronically or on paper with pre-paid postal return). Any links sent either by email or text to a questionnaire is unique to a participant and their timepoint/questionnaire in the study. Paper- or telephone-administered questionnaires may also be used where use of electronic means is not possible or suitable. If questionnaires are administered via paper or telephone, data will be entered into the study database by the central study team.

### Data collection

Baseline: Baseline sociodemographic, handedness, and injury data will be collected in the baseline CRFs. Participants will also be asked to complete the PRWE, PROMIS Physical Function (Upper Extremity), EQ-5D-5L, and SEE Scale.

Treatment logs: After the usual care or intervention (in addition to usual care) sessions the date, duration, session content, clinician profession and experience details, setting, mode of delivery, and the material and resources issued will be recorded on treatment logs.

Three- and six-month post-randomization follow-up: Participants will receive electronic or paper invites to complete questionnaires, which include the PRWE, PROMIS, EQ-5D-5L, SEE, exercise frequency, resource use, and adverse events. Reminders will be sent by email, post, and/or text message (according to the participant’s preference). If questionnaires are not completed, these will be followed by a phone call from the central study team if needed. Should data queries arise from participant-completed questionnaires, the central study team will attempt to contact the participant by telephone, email, or text message to resolve the query if it is not appropriate to be clarified with the clinical site team.

Six-month post-randomization follow-up muscle strength measurement: Participants who return the six-month follow-up questionnaires will then immediately be sent a dynamometer at six months’ follow-up with instructions on how to use the device and will have access to a video guide. Strength measurements will not be attempted if the six-month questionnaires are not returned, or are returned more than two weeks late. Participants will be contacted to offer support to do the assessments by a member of the research team via telephone or video call. If the strength measurements are not completed, the study team will attempt to contact participants as reminders. The participant will be provided with return packaging with freepost return so they can send the dynamometer back to the central study team.

### Communication with study participants by the CTU study team

Participants will be notified to complete study questionnaires by email or text message; where they have selected to receive postal questionnaires, these will be posted to the participant. Participants may be sent up to two reminder messages and/or emails/letters to complete their questionnaires. Participants who do not complete their study questionnaires may be telephoned to collect the data or request return of the questionnaire.

### Withdrawal

Withdrawal of consent means that a participant has expressed a wish to withdraw from the study altogether or from certain aspects of the study only. The type of withdrawal will be collected on the CRF labelled ‘withdrawal’.

Participants may also be withdrawn from the study (or aspects of the study) by their clinician if they believe this is necessary to safeguard the safety or wellbeing of the participant, including but not limited to ineligibility (either arising during the study or retrospectively having been overlooked at screening).

The withdrawal CRF should be completed to document the reasons for withdrawal, and state who made the decision to withdraw. Discussions and decisions regarding withdrawal should be documented in the participant’s medical notes. Investigators should continue to follow up any SAEs, and should continue to report any SAEs to resolution in the CRF in accordance with the safety reporting section.

Where a participant expresses a wish to withdraw from the study, the research team will determine which aspect(s) of the study the participant wishes to withdraw from. A participant may request to withdraw if they are no longer willing to complete study questionnaires. Participants who decide not to attend a supervised rehabilitation therapy session(s) will not be considered a study withdrawal.

Participants will not be asked to participate in the collection of remaining follow-up data after being withdrawn. The reason for withdrawal will be recorded on the study withdrawal CRF. Withdrawn participants will not be replaced, as we have allowed for possible withdrawals and loss to follow-up in the estimated sample size.

Completion of the withdrawal CRF by the site research team will trigger a notification to the central study office. Appropriate action will be taken by the study teams (centrally at the CTU and by the site research team at each participating site) to ensure compliance with the participant’s withdrawal request. This may include marking future CRFs as not applicable, and ensuring any relevant communications which the participant had consented to receive regarding their participation are no longer sent.

Data collected up to the point of withdrawal will be used/analyzed as explained in the PIS, unless the participant specifically requests otherwise.

## Blinding and code breaking

Table V provides an overview of the blinding status of all individuals involved in the conduct and management of the study.

**Table V.** Blinding status of those involved in study conduct and management.

Role in study	Blinding status	Additional information
Participants	Not blinded	It is not possible to blind due to nature of the intervention. Participants will be told their treatment allocation immediately after randomization.
Site research staff including PI	Not blinded	Not possible due to the nature of the intervention. Following randomization, an email will be sent to the PI and/or member of the site research team performing the randomization (as delegated) confirming treatment allocation.
CI	Blinded for those at sites other than their own, except for any SAE causality assessment	It is not possible to blind the CI as they may be the primary clinician for those participants recruited at their site, however they will be blinded to allocations for participants at other sites. In instances where serious adverse events are reported, the CI will become unblinded to complete the full causality assessment.
Database programmer	Not blinded	The database programmer is responsible for the management of the randomization system and the REDCap database and will have access to all unblinded datasets within both systems.
WISE study management staff within Oxford Clinical Trial Research Unit: Oxford Trauma	Not blinded	Study management staff within Oxford Clinical Trial Research Unit will not be blinded to treatment allocations as site staff may require support for randomization, or participants may contact the study team directly. SAE reports will also be handled by the study management team which will contain allocation information. Staff calling participants to collect outcome data will receive training and follow scripts to ensure a consistent approach.
Data management	Not blinded	Data management staff will have access to the unblinded datasets within the study randomization system and database to ensure data quality and undertake central monitoring activities.
Study statistician and senior study statistician	Not blinded	The study statistician and senior study statisticians will have access to treatment allocations or data needed for generating the trial oversight committee closed reports and the final analysis.

CI, Chief Investigator; PI, Principal Investigator; SAE, serious adverse event.

### Safety reporting period

Safety reporting for each participant will begin from randomization and will end when the participant has reached their final main follow-up timepoint, at six months post-randomization.

### Definitions

A distinction is drawn between serious and severe AEs. Severity is a measure of intensity whereas seriousness is defined using the criteria above. Hence, a severe AE need not necessarily be serious (Table VI).

**Table VI.** Definitions of adverse events.

AE	Any untoward occurrence in a clinical study participant. Note: an AE can therefore be any unfavourable and unintended sign (including an abnormal laboratory finding, for example), symptom, or disease temporarily associated with the study procedures, whether or not considered related to the procedures.
Related AE	An event that resulted from administration of any of the research procedures
SAE	An AE that: results in death is life-threatening* requires hospitalization or prolongation of existing hospitalization results in persistent or significant disability or incapacity is a congenital anomaly or birth defect; or is otherwise considered medically significant by the Investigator†
Unexpected related SAE	A SAE related to the study (i.e. resulted from administration of any of the research procedures) and is unexpected (not listed in the protocol as an expected occurrence).

* Participant was at risk of death at the time of the event; it does not refer to an event which hypothetically might have caused death if it were more severe.

† Medical events that may jeopardize the participant or may require an intervention to prevent one of the above characteristics/consequences.

AE, adverse event; SAE, serious adverse event.

### Expected adverse events

Foreseeable AEs include:

- increase in pain lasting more than one week
- treatment-related exacerbations of other medical conditions that do not meet the definition of serious (e.g. angina after exertion)
- development of complex regional pain syndrome
- surgery to the injured wrist (unless an adverse event directly related to the exercise intervention, in which case this would be an SAE)
- development of carpal tunnel syndrome requiring medical intervention (corticosteroid injection or surgery)
- wound complication in the injured wrist

### Reportable AEs/SAEs

Foreseeable SAEs and adverse events not defined as serious that are related to the interventions will be recorded by participants or site staff, but will not need to be reported immediately. These events will be recorded on patient-reported questionnaires or by the site investigators in the ‘complications’ CRF if they become aware of such an event.

### Non-reportable AEs/SAEs

AEs that are unrelated to the intervention will not be reported. AEs deemed related to the intervention that do not meet the SAE definition and are not classed as foreseeable (such as discomfort during performance of exercises) will also not be reported.

### Procedure for collecting safety events from sites/participants

These events will be recorded on patient-reported questionnaires and by the site investigators in the ‘complications’ CRF if they become aware of such an event.

### Reporting of SAEs from sites to the CTU study team

Only SAEs considered by the site investigator to be related (possibly, probably, or definitely) to the study intervention/any of the research procedures will be reported immediately to the central study team.

SAEs will be reported by the site research team using the SAE form within the REDCap study database within 24 hours of becoming aware of the event. The CTU is automatically notified of the SAE report through the database. A paper SAE form should be used as a back-up if the SAE form is not available electronically. This should be emailed within 24 hours of becoming aware of the event. The central study team will acknowledge receipt of any SAEs reported via email within one working day and provide the site with a unique SAE log number.

The PI (or delegated individual) is responsible for assessing all reported SAEs for seriousness, causality, and expectedness.

Relatedness/causality: The assessment of ‘relatedness’ to the study intervention is the responsibility of the PI at site or an agreed designee, according to the definitions outlined in Table VII.

**Table VII.** Relatedness/causality.

Relationship to intervention	Attribution (causality)	Description
Unrelated	Unrelated	The AE is clearly NOT related to the intervention
	Unlikely	The AE is doubtfully related to the intervention
Related	Possible	The AE may be related to the intervention
	Probable	The AE is likely related to the intervention
	Definite	The AE is clearly related to the intervention

AE, adverse event.

For the purpose of safety reporting, the intervention is defined as the therapy sessions, whole exercise programme, and the workbook/intervention website.

### Review of SAEs by the sponsor/CTU nominated person

An appropriately qualified person will review the SAE and raise any queries with the reporting site. If the site has not provided an assessment of causality and has not responded to the query, it will be assumed that the event reported is related to the study procedures/intervention. The site will be encouraged to respond and if a response is not provided the CI will be consulted by the CTU and the CTU will complete the sponsor part of the SAE report.

### Reporting of SAEs to the research ethics committee

All intervention/study procedure-related SAEs will be recorded in the study database. All SAEs that are assessed as related and unexpected will be submitted to the research ethics committee (REC) within 15 days of the CTU/Sponsor becoming aware of the event.

### Follow-up of SAEs

If the SAE is an unexpected related event, then follow-up information must be provided as requested by the study office. A follow-up report must be completed when the SAE resolves, is unlikely to change, or when additional information becomes available.

### Pregnancy

If a participant does become pregnant during their participation in the study, it does not need to be reported due to the nature of the intervention as concluded in the risk assessment of the study.

## Statistical considerations

### Statistical analysis plan

The statistical aspects of the study are summarized here, with details fully described in a statistical analysis plan (SAP) that was drafted early in the study and finalized prior to the final analysis data lock (see the full SAP in the Supplementary Material). The SAP was developed by the study statistician (IM) in accordance with the current OCTRU standard operating procedures (SOPs). An independent trial oversight committee (TOC) will review and, if necessary, provide input into the SAP. Any deviation(s) from the original SAP will be described in the final statistical report.

### Sample size/power calculations

The sample size calculation requires 588 participants randomized across the two treatment groups, with the PRWE at six months as the primary outcome. This calculation is based on a clinically meaningful difference of six points in the PRWE,^7^ a SD of 20, power of 90%, an α error of 0.05 (two-sided) and assuming a 20% loss to follow-up of primary outcome data. A mean difference in the PRWE of six points is just above the amount achieved if all the participants in one group indicated they had one-degree better response to any of the PRWE’s constituent questions (e.g. one degree less difficulty in turning a doorknob) than the other group (each degree in response contributes five points to the overall score). The previous NIHR-funded wrist fracture clinical trials conducted by the team have used six points as the minimal clinically important difference. The SD of 20 was based on the pooled PRWE data from the WISE feasibility phase (NIHR200458).

### Description of statistical analysis

Results will be reported in line with the CONSORT statement and any appropriate extensions, and will be described fully in a separate SAP. A single final unblinded statistical analysis will take place after all follow-up has been completed, and sufficient time has been allowed for data collection and cleaning.

It is anticipated that all statistical analysis will be undertaken using Stata (StataCorp, USA) or other validated statistical software.

All available data from both treatment arms will be analyzed. This analysis will include data collected as part of the feasibility phase (NIHR200458) for the usual care and the supervised exercise groups. Descriptive statistics will be used to describe the demographics for the two intervention groups, reporting means and SDs or medians and IQRs, as appropriate, for continuous variables and numbers and percentages for binary and categorical variables. Statistical summaries and graphical plots will be presented for the primary and secondary outcome measures. The primary analysis will use the randomized ‘intention-to-treat’ (ITT) population, analyzing participants with available outcome data in their randomized groups, regardless of adherence to their allocated intervention. All outcomes will be presented with effect sizes and 95% CIs and will be assessed with 5% level of significance (except for subgroup analyses). p-values will be reported with up to three decimal places.

Primary outcome: The PRWE score at six months is the primary outcome and will be compared between treatment groups as the dependent variable in a mixed-effects linear regression model including outcome information at three months. This model will adjust for baseline PRWE score and the stratification factors (recruitment centre and initial fracture management (surgical compared with non-surgical)). Random effects will be included to account for heterogeneity in response due to recruitment centre and for repeated observations within participant, and the adjustment variables will be incorporated as fixed effects. The treatment effect will be based on the adjusted mean differences at six months which will be reported together with their 95% CIs. To explore the effect of compliance on the treatment effect a complier average causal effect (CACE) analysis will be undertaken. The definition of compliance with the intervention will be finalized in the SAP.

Secondary outcome(s): Secondary clinical outcomes and patient-reported outcomes will be analyzed using generalized linear models, with model adjustment similar to the primary analysis described above. In addition to the analysis of the secondary outcomes, the number of unexpected serious adverse events and complications will be reported by type for each intervention group and the proportion of participants with at least one event or one complication will be compared. The details of the adverse event or complication together with information on the timing of the events will be presented.

### Inclusion in analysis

The principal analysis will be performed on the ITT population, analyzing participants with available outcome data in their randomized groups, regardless of adherence.

### Subgroup analysis

Prespecified subgroup analyses will be conducted by utilizing subgroup-by-treatment interactions for prognostic factors such as initial fracture management, with full details finalized in the SAP. Results will be displayed and viewed as exploratory.

### Interim analyses

The main outcomes will be analyzed as stated in the SAP once the study follow-up has been completed. There are no plans for carrying out any formal interim analysis of the main outcomes of the study. Interim analyses of the efficacy outcomes are not planned and will be performed only if requested by the TOC.

Stopping rules: As no formal interim analyses are planned, no stopping rules have been incorporated into the study design. The TOC will review the accumulating data at regular intervals and may recommend pausing or stopping the study in the event of safety concerns.

### Procedure for accounting for missing data

The procedure for handling spurious or missing data will be described in the SAP. Missing data will be minimized by careful data management. Missing data will be summarized and patterns analyzed, with reasons given where available. All data collected on data collection forms will be used, since only essential data items will be collected. No data will be considered spurious in the analysis, since all data will be checked and cleaned before analysis. The nature and mechanism for missing variables and outcomes will be investigated and multiple imputation will be used if appropriate. Multiple imputation also assumes a missing-at-random mechanism and is therefore not expected to add value to the primary analysis model.^47^

## Health economics

A health economist has reviewed the data collection to ensure that a minimum set of economic data are collected. Further funding will be obtained to allow an accompanying economic evaluation to take place (see Supplementary Material for the Health Economic Analysis Plan).

## Data management

The data management aspects of the study are summarized here with details fully described in the study-specific data management plan.

### Case report forms

The investigator and study site staff will ensure that data collected on each participant is recorded in the CRF as accurately and completely as possible. Details of all protocol evaluations and investigations must be recorded in the participant’s medical record for extraction onto the CRF. All investigator observations will be transcribed into the CRFs from the relevant source data held in the site medical record(s).

All documents will be stored safely in confidential conditions. On all study-specific documents, other than the signed consent, the participant will be referred to by the study participant number/code, not by name.

Source data to be recorded directly on the CRFs: entries will be considered source data if the CRF is the site of the original recording (e.g. there is no prior written or electronic record of data). There will be no non-CRF data in this study.

### Access to data

To ensure compliance with regulations, direct access will be granted to authoried representatives from the Sponsor and host institution to permit study-related monitoring, audits, and inspections. The data submitted by study participants directly via the study database (i.e. electronic participant-reported outcomes) will also be made available to the participating site that recruited the participant; this is detailed within the PIS so that participants are aware of who will have access to this data.

Members of the study team will only be able to access data that they need to, based on their roles and responsibilities within the study.

### Data recording and record keeping

The case report forms have been designed by members of the study management team which will include the CI, study statistician(s), and study manager.

Data will, wherever possible, be collected in electronic format with direct entry onto the study database by site staff or participants. Electronic data collection has the major advantage of building ‘data logic’ into forms, minimizing missing data, data input errors, and ensuring the completeness of consent and assent forms. REDCap is a secure, web-based application designed to support data capture for research studies, providing: 1) an intuitive interface for validated data entry; 2) audit trails for tracking data manipulation and export procedures; 3) automated export procedures for seamless data downloads to common statistical packages; and 4) procedures for importing data from external sources.

All data entered will be encrypted in transit between the client and server. All electronic patient-identifiable information, including electronic consent forms, will be held on a server located in an access-controlled server room at the University of Oxford. The database and server are backed up to a secure location on a regular basis. Details of the data collected, where they are stored, and who has access to them, along with a fair processing statement, will be available to the participants within the study PIS.

Personal identifiable data will be kept separately from the outcome data obtained from/about the patients. Patients will be identified by a study ID only. Direct access to source data/documents will be required for study-related monitoring and/or audit by the Sponsor, research team, or NHS Trust or regulatory authorities as required.

Data captured during phone calls to participants or from paper-based study questionnaires returned to the study office will be entered into the study database by suitably trained central study office staff. Full details of this process will be recorded in the data management plan. Identifiable data will only be accessible by members of the research team with a demonstrated need (managed via access controls within the application) and only used to communicate with the participant (e.g. sending follow-up reminders for online form completion or telephone follow-up).

Audio recordings of treatment will be made digitally on password-protected devices. They will be stored on secure servers at the University of Oxford, identified by a trial ID and/or initials only and will only be accessible to the CI and those members of the Oxford research team who have been authorized to do so by the CI. The audio recordings will be retained for 12 months after being received and analyzed as part of intervention quality assurance and then deleted. It is necessary to retain the recordings for this period as they are the source data and help us to assess treatment delivery. Access to them is required in case these are needed to refer back to these during intervention reporting.

### Electronic transfer of data

Any electronic transfer of data during the course of the study will be strictly controlled in accordance with the OCTRU SOPs for secure information/data transfer.

## Quality assurance procedures

A rigorous programme of quality control will be implemented. The study management group will be responsible for ensuring adherence to the study protocol at the study sites. Quality assurance (QA) checks will be undertaken by OCTRU to ensure integrity of randomization, study entry procedures, and data collection. The OCTRU has a QA team who will monitor this study by conducting audits of the TMF. Furthermore, the processes of obtaining consent, randomization, registration, provision of information, and provision of treatment will be monitored by the central study team. Additionally, the study may be monitored, or audited by Sponsor or host sites in accordance with the current approved protocol, GCP, relevant regulations, and standard operating procedures. A study-specific data management and monitoring plan will be in place prior to the start of the study.

### Risk assessment

This protocol is designed to deliver a risk-adapted approach to conducting the research. A risk assessment has been conducted and a monitoring plan will be prepared before the study opens. The known and potential risks and benefits to participants have been assessed in comparison to those of standard of care. A risk management strategy is in place and will be reviewed and updated as necessary throughout the study or in response to outcomes from monitoring activities. Monitoring plans will be amended as appropriate.

### Study monitoring

Monitoring will be performed by the central study team according to a study-specific monitoring plan. Data will be evaluated for compliance with the protocol, completeness, and accuracy. The investigator and institutions involved in the study will permit study-related monitoring and provide direct on-site access to all study records and facilities if required. They will provide adequate time and space for the completion of monitoring activities.

Study sites will be monitored centrally by checking incoming data for compliance with the protocol, consistency, completeness, and timing. The CRF data will be validated using appropriate set criteria, range, and verification checks. The study site must resolve all data queries in a timely manner. All queries relating to key outcome and safety data, and any requiring further clarification, will be referred back to the study site for resolution.

Study sites will also be monitored remotely and/or by site visit, as necessary, to ensure their proper conduct of the study. Study office staff will be in regular contact with site personnel to check on progress and deal with any queries that they may have. Any monitoring reports/data discrepancies will be sent to the site in accordance with OCTRU SOPs and the study monitoring plan. The investigator is expected to action any points highlighted through monitoring and must ensure that corrective and preventative measures are put into place as necessary to achieve satisfactory compliance, within 28 days as a minimum, or sooner if the monitoring report requests.

Intervention delivery will be monitored periodically to ensure fidelity. Site visits and/or audio recording of interventions will be conducted. Permission will be sought from the trial participants to observe or record treatment sessions. Verbal consent will be provided and recorded.

CRFs will also be used to monitor intervention fidelity. Data will be collected on intervention content delivery and number of treatment sessions attended to facilitate monitoring and reporting. The sites will regularly receive feedback from quality activities to help maintain and improve fidelity.

### Audit and regulatory inspection

All aspects of the study conduct may be subject to internal or external quality assurance audit to ensure compliance with the protocol, GCP requirements, and other applicable regulation or standards. Such audits or inspections may occur at any time during or after the completion of the study. Investigators and their host institution(s) should understand that it is necessary to allow auditors direct access to all relevant documents, study facilities and to allocate their time and the time of their staff to facilitate the audit visit. Anyone receiving notification of an audit that will (or is likely to) involve this study must inform the study office without delay.

### Study committees

Trial management group (TMG): This will be established for the study and operate in accordance with a study-specific TMG charter. The TMG will manage the trial, including the clinical and practical aspects and will meet approximately monthly to assess progress. Other specialities/individuals will be invited as required for specific items/issues.

Data and safety monitoring committee (DSMC): There is no DSMC to be convened for this study; instead, a TOC is to be formed to take the role that a DSMC and TSC would usually undertake.

Trial steering committee (TSC): There is no TSC to be convened for this study; instead, a TOC is to be formed to take the role that a DSMC and TSC would usually undertake.

TOC: The TOC, which includes independent members, provides overall supervision of the trial on behalf of the funder. Its terms of reference will be drawn up in a TOC DAMOCLES-based charter, which will outline its roles and responsibilities. Meetings of the TOC will take place at least once a year during the recruitment period. An outline of the remit of the TOC is to:

- Monitor and supervise the progress of the study towards its interim and overall objectives.
- Review accruing data, completeness, and blinded summaries if required, and assess the screening algorithm against the eligibility criteria.
- Consider emerging evidence from other related studies or research.
- Review any safety issues and make recommendations whether the protocol should be amended to protect patient safety.
- Inform the funding body on the progress of the study.

The TOC will include at least one PPI representative as an independent member. Full details including names will be included in the TOC charter. Recommendations of the TOC will be discussed between the CI and the sponsor.

## Identification and management of participating sites

### Identification of recruitment sites

Recruitment sites will be selected based on suitability to conduct the study. Potential sites will be invited to complete a site feasibility questionnaire (SFQ) which will be used by the TMG/coordinating centre to assess suitability of the site for the study; the suitability assessment will primarily be based on the resources available at site and the feasibility of meeting recruitment targets.

### Study site responsibilities

The PI, or lead clinician for the study site, has overall responsibility for the conduct of the study but may delegate responsibility where appropriate to suitably experienced and trained members of the site research team. All members of the site research team must complete a delegation log provided by the central study team prior to undertaking any study duties. The PI must counter sign and date each entry in a timely manner, authorizing staff to take on the delegated responsibilities.

### Study site set up and activation

The PI leading the participating study site is responsible for providing all required core documentation. Mandatory site training which is organized by the study office must be completed before the site can be activated. Training in the study processes will be administered at site initiation visits, delivered either in person or online by the central study team. The study office will check to confirm that the site has all the required study information/documentation and is ready to recruit. The site will then be notified once they are activated on the study database and are able to begin recruiting participants.

### Study documentation

The study office will provide an electronic ISF to each participating site containing the documents needed to conduct the study. The study office must review and approve any local changes made to any study documentation including patient information and consent forms prior to use. Additional documentation generated during the course of the study, including relevant communications must be retained in the site files as necessary to reconstruct the conduct of the study.

## Ethical and regulatory considerations

The investigator will ensure that the study is conducted in accordance with the principles of the Declaration of Helsinki.^48^ The investigator will also ensure that the study is conducted in accordance with relevant regulations and with the principles of good clinical practice.

### Ethical conduct of the study and ethical approvals

The protocol, PIS, ICF, and any other information that will be presented to potential study participants (e.g. advertisements or information that supports or supplements the informed consent process) will be reviewed and approved by an appropriately constituted, independent REC.

### NHS research governance

Once HRA & Health and Care Research Wales (HCRW) and/or Research & Development study-wide review in Scotland and Northern Ireland approval is in place for the study, sites will confirm capability and capacity to participate in the study.

### Protocol amendments

All amendments will be generated and managed according to the OCTRU SOPs to ensure compliance with applicable regulation and other requirements. Written confirmation of all applicable REC and local approvals must be in place prior to implementation by Investigators as applicable for the amendment type. The only exceptions are for changes necessary to eliminate an immediate hazard to study participants (see below).

It is the investigator’s responsibility to update participants (or their authorized representatives, if applicable) whenever new information (in nature or severity) becomes available that might affect the participant’s willingness to continue in the study. The Investigator must ensure this is documented in the participant’s medical notes and the participant is re-consented if appropriate.

### Protocol compliance and deviations

Protocol compliance is fundamental to Good Clinical Practice (GCP). Prospective, planned deviations or waivers to the protocol are not allowed. Changes to the approved protocol need prior approval unless for urgent safety reasons.

A study-related deviation is a departure from the ethically approved study protocol or other study document or process or from GCP or any applicable regulatory requirements. Deviations from the protocol will be captured within the study database either using a protocol deviation form or via suitably designed fields within the CRF which will be extracted from the study database and reviewed regularly by the TMG. Deviations will be handled and reviewed in a timely manner in accordance with a study-specific Data Management and Monitoring Plan.

The investigator must promptly report any important deviation from GCP or the protocol to the study office. Examples of important deviations are those that might impact on patient safety, primary/secondary endpoint data integrity, or be a possible serious breach of GCP.

### Urgent safety measures

The sponsor or investigator may take appropriate urgent safety measures to protect study participants from any immediate hazard to their health or safety. Urgent safety measures may be taken without prior authorization. The study may continue with the urgent safety measures in place. The investigator must inform the study office immediately if the study site initiates an urgent safety measure. The notification must include the date of the urgent safety measure, who took the decision, and why the action was taken.

The investigator will provide any other information that may be required to enable the study office to report and manage the urgent safety measure in accordance with the current regulatory and ethical requirements for expedited reporting and close out. The study office will follow written procedures to implement the changes accordingly.

### Temporary halt

The sponsor and investigator reserve the right to place recruitment to this protocol on hold for short periods for administrative reasons or to declare a temporary halt. A temporary halt is defined as a formal decision to:

- interrupt the treatment of participants already in the study for safety reasons;
- stop recruitment on safety grounds; or
- stop recruitment for any other reason(s) considered to meet the substantial amendment criteria, including possible impact on the feasibility of completing the study in a timely manner.

The study office will report the temporary halt via an expedited substantial amendment procedure. The study may not restart after a temporary halt until a further substantial amendment to reopen is in place. If it is decided not to restart the study, this will be reported as an early termination.

### Serious breaches

A ‘serious breach’ is a breach of the protocol or of the conditions or principles of GCP which is likely to affect to a significant degree:

- the safety or physical or mental integrity of the study subjects; or
- the scientific value of the research.

Investigators must notify the study office within one working day if any serious breach of GCP is suspected. The study office will review the event and, if appropriate, will report a serious breach to the REC and the NHS host organization within seven days of the study office becoming aware of the breach.

### Transparency in research

Prior to the recruitment of the first participant, the study will be registered on a publicly accessible database (ISRCTN), which will be kept up to date during the study, and we aim for results to be uploaded to the registry within 12 months of the end of the study declaration subject to publication of the main trial results in a scientific journal. A final report will be submitted to the REC containing a lay summary of the study results which will be published on the HRA website. The results of the study will be published and disseminated.

Social media (e.g. X feeds) may be used to promote the study, and acknowledge when milestones are met (e.g. sites open to recruitment, first recruitment by a site).

## Participant confidentiality

### Collection and use of personal identifiable information

Contact details (e.g. email addresses/postal addresses/phone number) will be collected in this study for the following purposes, and where an activity is optional, only with the specific consent of the participant:

- Sending of follow-up questionnaires and any reminder messages
- Sending text messages regarding follow-up questionnaires
- Sending a copy of the completed consent form by email (for any participants that consent electronically and wish to receive a copy by email)
- Enabling access to the intervention website (to those allocated to supervised exercise)
- Sending of dynamometers direct to participants’ homes
- Collection of NHS number/CHI number

The PIS explains what contact details will be collected and how these will be used; explicit consent will be obtained for this. Site staff at participating sites will ensure that contact details for study participants are up to date when participants attend for study visits. Where remote eConsent is used, participants will be asked to give their permission verbally for a link to the consent documentation to be sent to their email address or an email address they provide.

### Storage and use of personal data

Personal data during the study will be stored and used in accordance with the OCTRU SOPS for confidentiality, protection, and breach of personal data in relation to research subjects. This ensures that all personal data collected during the study is recorded, handled, and stored in such a way that is satisfies the requirements of the UK General Data Protection Regulation (GDPR) and requires data to be anonymized as soon as it is practical to do so.

All electronic patient-identifiable information will be held on a secure, password-protected database, accessible only to authorized personnel. Paper forms with patient-identifiable information will be held in secure, locked filing cabinets within a restricted area. The processing of the personal data of participants will be minimized wherever possible by the use of a unique participant study number on study documents and any electronic databases.

Personal data on all documents will be regarded as confidential. The study staff will safeguard the privacy of participants’ personal data.

The use of all personal data in the study will be documented in a study-specific data management and sharing plan, which details what and where personal data will be held, who will have access to the data, when personal data will be anonymized, and how and when it will be deleted. The investigator site will maintain the patients’ anonymity in all communications and reports related to the research.

Data breaches will be highlighted to the relevant site staff and reported as required by the UK GDPR and Data Protection Act 2018. This will also be deemed a protocol deviation.

### Access to participants’ personal identifiable data during the study

Access to participants personal identifiable data will be restricted to individuals authorized to have access. This includes: 1) members of the research team at participating study sites with delegated responsibility by the site PI; and 2) members of the central study team involved in the conduct/management of the study where this is necessary for their role.

Research staff who are not part of the participant’s direct healthcare team will not have access to personal identifiable data until the participant has given their consent to take part in the study, or the participant has indicated to their direct healthcare team that they wish to be contacted by a member of the site research team – permission for this will be recorded in the participant’s medical notes. The PIS clearly describes who will have access to the participants’ personal identifiable data during the study and explicit consent will be obtained from study participants for such access.

Participants will be asked to consent to relevant sections of their medical notes and data collected during the study being looked at by individuals from the University of Oxford, from regulatory authorities (and from the NHS Trust(s)), where it is relevant to their taking part in this study; only authorized individuals will be granted access where this is necessary for their role.

### Destruction of personal identifiable data

Explicit consent for the storage and use of personal identifiable data (which includes consent forms) will be obtained from participants as detailed in the PIS and ICF. Personal identifiable data will be destroyed as soon as it is no longer required; the timepoint for this destruction is detailed in the study data management plan and is in accordance with OCTRU standard operating procedures which comply with the UK GDPR.

### Participant identification log

The site research team must keep a separate log of enrolled patients’ personal identification details as necessary to enable them to be tracked. These documents must be retained securely, in strict confidence. They form part of the investigator site file and are not to be released externally.

## Public and patient involvement

### PPI in study design and protocol development

The design of this study was supported by four PPI representatives who attended a workshop. Their feedback from their own experiences of distal radius fracture and other injuries was that after the acute phase the feeling of weakness and lack of confidence in the limb was a common experience and a barrier to resistance exercise and were supportive of the value of further research in this area. The PPI group highlighted the challenges of motivation when exercising independently and that the quality of information would be crucial to success. The PPI feedback directly informed the design of the WISE intervention and influenced the trial design and choice of outcomes.

The PPI members of the management group have specifically been involved in producing the developed:

- trial patient information
- CRFs
- recruitment and consenting procedures
- patient leaflets
- intervention workbooks and website
- dissemination plan

When developing the intervention workbooks and websites, there were several rounds of user testing, including four other PPI representatives. This phase of intervention development resulted in amendments to the materials to improve usability and altering the written and verbal information to make them accessible to people with a wide range of literacy levels.

### PPI during the study

Two members of the PPI group are part of the research team as co-applicant members of the TMG (JG, RG), attending monthly meetings. The TOC will also have a PPI member.

### PPI during dissemination of study results

The PPI co-applicants will lead the WISE dissemination to patients/service users, carers, and the wider population directly through their extensive network of patient advocacy organizations and charities. They will help generate a plain language summary for patients and the public, which will also be used as the basis for an explainer animation video.

## Sponsorship, finance, and insurance

### Sponsorship

The sponsor will provide written confirmation of sponsorship. A separate study-specific delegation of responsibilities will outline the responsibilities of the CI, sponsor, and the Oxford Clinical Trials Research Unit.

### Insurance

The Sponsor (University of Oxford) has a specialist insurance policy in place which would operate in the event of any participant suffering harm as a result of their involvement in the research (Newline Underwriting Management, at Lloyd’s of London). NHS indemnity operates in respect of the clinical treatment that is provided.

## Contractual arrangements

Appropriate contractual arrangements will be put in place with all third parties. This study is subject to the Sponsor’s policy requiring that written contracts/agreements are agreed formally by the participating bodies as appropriate. The Sponsor will also set up written agreements with any other external third parties involved in the conduct of the study as appropriate.

## Publication and dissemination

The Sponsor will retain ownership of all data arising from the study. Publication and dissemination of study results will be in accordance with OCTRU SOPs and irrespective of study findings. All data will be presented such that no individual participants can be identified.

The study protocol will be published in an open-access peer-reviewed journal in accordance with the Standard Protocol Items: Recommendations for Interventional Trials statement (SPIRIT).^49^ The study results will be published in an open-access journal, in accordance with the National Institue for Health and Care Research (NIHR)’s policy on open-access research.^50^ The study will be reported following the CONSORT guideline including any applicable extensions to this. The Template for Intervention Description and Replication (TIDieR) statement will be used for reporting the intervention.^51^ The SAP will be published in an open-access peer-reviewed journal before any primary outcome analysis.

### Dissemination of study results to participants

A summary of the study results for study participants will be written collaboratively with clinicians and patient representatives and distributed accordingly. The PIS includes a link to the study website where participants will be advised that the results will be published. Newsletters, X, etc, will also be used to ensure the results of the study are communicated to the wider community once they are available.

Dissemination of results will include the following methods:

- Conference: The results of this study will be disseminated to the clinical community via presentations at national and international conferences. Traditional conference dissemination will focus on presentations to include the key professional stakeholders.
- Publications: Results will usually be published in peer-reviewed journals. Where possible, plain English summaries will be published alongside the full paper, along with links to other digital media on the study website to explain the study result in an accessible format – i.e. an explainer video and infographic.
- Public dissemination: To ensure a broad campaign we will target a range of social media outlets (this may include an explainer video and infographic). The wider public will be alerted via links with relevant organizations/charities, and the research media offices. Engagement with the NIHR Dissemination Centre will also be sought, to ensure global awareness of study findings. Moreover, the University of Oxford and Oxford University Hospitals NHS Trust have professional communication officers. It is anticipated that together these individuals, and NIHR equivalents, will agree upon effective communication strategies including coordinated press releases, interviews, etc.
- Guidelines: The aim of this study is to inform updates to the National Institute for Health and Care Excellence (NICE) guideline NG38: Fractures (non-complex)^52^ and the Best Practice for Management of Distal Radial Fractures guidance from the British Orthopaedic Association and British Society for Surgery of the Hand.^13^

### Authorship

Authorship of any publications arising from the study will be determined in accordance with the International Committee of Medical Journal Editors (ICMJE) guidelines and any contributors acknowledged accordingly. All publications arising from this study must acknowledge the contribution of participants, funder(s), OCTRU, Oxford Trauma and Emergency Care, and the sponsor.

### Intellectual property

Ownership of intellectual property (IP) generated by employees of the university vests in the university. The university will ensure appropriate arrangements are in place as regards any new IP arising from the study.

## Archiving

### Minimum mandatory archiving period

It is the University of Oxford’s policy to store data for a minimum of three years following publication. Investigators may not archive or destroy study essential documents or samples without written instruction from the study office. The minimum mandatory archiving period for essential study documents for this study is three years following publication.

### Archiving responsibilities/procedure

During the study and after study closure, the investigator must maintain adequate and accurate records to enable the conduct of a clinical study and the quality of the research data to be evaluated and verified. All essential documents must be stored in such a way that ensures that they are readily available upon request for the minimum period, as specified above.

CTU Trial Master File: All paper and electronic data including the TMF and study database will be retained and archived in accordance with OCTRU’s standard operating procedures which are compliant with the UK GDPR.

Investigator site file and participant medical records: The investigator site files will be archived at the participating site. The medical files of study participants must be retained for the mandatory archiving period stated above and in accordance with the maximum period of time permitted by the participating site. Sites should comply with the documentation retention specified in the clinical trial agreements (or equivalent) issued by the study sponsor.

### Retention of datasets

Study data and associated metadata will be held electronically in a suitable format in a secure server area maintained and backed up to the required standard. Access will be restricted to the responsible Archivist and will be controlled by a formal access request. On completion of the mandatory archiving period, the TMF and associated archived datasets will be destroyed or transferred as appropriate, according to any data sharing requirements.

## Data sharing

The study statistician may retain copies of anonymized datasets for the purpose of data sharing in accordance with the study data sharing plan. Requests for data (anonymized trial participant level data) will only be provided at the end of the trial to external researchers who provide a methodologically sound proposal to the trial team (and who will be required to sign a data sharing access agreement with the Sponsor) and in accordance with the NIHR guidance. After the end of the study, an anonymized study dataset will be created and stored for as long as it is useful and may be shared with other researchers (upon request). Participant consent for this is included in the informed consent form for the study.

### Retention of anonymized datasets

Upon completion of the study, anonymized research data may be shared with other organizations on request to the CI and in accordance with the data sharing policies of OCTRU, the sponsor and funder(s), as outlined in the PIS.

**Take home message**

- The WISE (Wrist Injury Strengthening Exercise) study is a multicentre, randomized controlled trial evaluating the effectiveness of a therapist-supervised exercise programme, compared to usual care advice, in improving pain and function after distal radius fractures in adults aged 50 years and over.

- The study aims to provide robust evidence to support patients and health professionals with decisions about post-fracture rehabilitation and inform updates to relevant clinical guidelines.
